# In Vitro Antibacterial Activity of *Hibiscus sabdariffa* L. Phenolic Extract and Its In Situ Application on Shelf-Life of Beef Meat

**DOI:** 10.3390/foods9081080

**Published:** 2020-08-08

**Authors:** Ana Selene Márquez-Rodríguez, Susana Nevárez-Baca, Julio César Lerma-Hernández, León Raul Hernández-Ochoa, Guadalupe Virginia Nevárez-Moorillon, Néstor Gutiérrez-Méndez, Laila Nayzzel Muñoz-Castellanos, Erika Salas

**Affiliations:** Facultad de Ciencias Químicas, Universidad Autónoma de Chihuahua, Chihuahua 31125, Mexico; anaselene.marquez@gmail.com (A.S.M.-R.); snebac@hotmail.com (S.N.-B.); julioclhz07@gmail.com (J.C.L.-H.); lhernandez@uach.mx (L.R.H.-O.); vnevare@uach.mx (G.V.N.-M.); ngutierrez@uach.mx (N.G.-M.); lmunoz@uach.mx (L.N.M.-C.)

**Keywords:** phenolic compounds, hibiscus flower, antimicrobial, shelf-life extension, beef, meat

## Abstract

Compounds from spices and herbs extracts are being explored as natural antibacterial additives. A plant extract used in traditional folk medicine is *Hibiscus sabdariffa* L., also known as Roselle. Therefore, the potential use of a phenolic hibiscus extract as antibacterial or natural food preservative was analyzed in vitro and in situ. A phenolic extract was obtained from hibiscus calyces and fractionated, and then the fractions were tested against foodborne pathogen bacteria. Liquid–liquid extraction and solid-phase extraction were used to fractionate the hibiscus extract, and HPLC was employed to analyze the fractions’ phenolic composition. Minimum bactericidal concentration (MBC) and minimal inhibitory concentration (MIC) were calculated for brute hibiscus phenolic extract, each of the fractions and pure commercial phenolic compounds. Bacteria tested were *Escherichia coli*, *Salmonella enterica* serovar Typhimurium, *Staphylococcus aureus*, *Listeria monocytogenes* and *Bacillus cereus*. The fraction obtained after liquid–liquid extraction presented the best performance of MBC and MIC against the bacteria tested. Furthermore, a hibiscus ethanolic extract was employed as a natural preservative to extend the shelf-life of beef. Microbiological, color and sensory analyses were performed to the meat during the shelf-life test. The application of the phenolic hibiscus extract also showed an increase of the duration of the meat`s shelf life.

## 1. Introduction

Food technologists are proposing alternatives to synthetic preservatives for the containment of foodborne pathogens; compounds from spices and herbs extracts are being explored as natural antibacterial additives. Phenolic compounds may contribute to the protective effect of spices and herbs extracts. Nowadays, the production of good quality food is a main goal amid the different production phases, from the grower to the table. Food safety has two main concerns. The first one is food spoilage, caused by microorganisms, which affects the nutraceutical and organoleptic characteristics and dramatically reduces shelf-life. The second and more important concern is posed by diseases (food poisoning) caused by foodborne pathogen bacteria. These two main concerns have been avoided by employing food preservatives, which have helped to extend shelf life and reduce the risk of disease. However, extended use of antibiotics in recent years has given some strains the resistance to commercial antibiotics, causing a health-related problem, since foodborne pathogens might survive to higher doses of antibacterial additives [1].

Otherwise, the resistance to commercial antibacterial agents could be diminished by employing natural preservatives, which do not create resistance in bacteria strains as synthetics ones synthetic antibacterial agents might. In both cases, putrefaction and diseases have been avoided since ancient times by using natural preservatives [2,3]. These natural preservatives consist of herbs, spices and essential oils, which include clove, garlic, cinnamon, oregano and onion, to name a few [4]. Besides, natural preservatives could present a synergistic effect with antibiotics over some resistant strains [5].

*Hibiscus sabdariffa* L. is a bush from the Malvaceae family, which grows in tropical and sub-tropical climates. The crop was possibly native to Asia and could have been scattered to Africa and then to America in the colonial period. Nowadays, hibiscus is widely cultivated throughout the world, where the principal producers are China, India, Malaysia, Sudan and Mexico. The plant is known as roselle, hibiscus, flor de jamaica, red sorrel or karkadé. Hibiscus calyces are used as a beverage in fermented drinks, jam, jellies or natural colorants. Besides, hibiscus infusion has significant use in folk medicine as a diuretic and as fever and hypertension reducer, or to improve digestion [6,7,8]. In addition, hibiscus has been studied for its antioxidant and antibacterial properties. The antibacterial activity in hibiscus, as in other spices and herbs, is related to its phenolic content, the major components on the extract [6]. Principal polyphenols with a known antibacterial activity are gallic acid, catechin, epicatechin, chlorogenic acid, protocatechuic acid and hydroxycinnamic acids [1,9,10]. Specifically, the antibacterial activity of roselle extract has been studied over foodborne pathogen bacteria, either in vitro and in a food model [11,12,13] Common foodborne pathogen bacteria strains tested are *Escherichia coli*, *Staphylococcus aureus*, *Salmonella Typhimurium*, *Listeria monocytogenes*, *Bacillus cereus*, *Shigella flexneri* and *Pseudomonas aeruginosa* [1,2,9,14,15,16]. 

The purpose of this study was to research the in vitro antibacterial activity of the phenolic extract of *Hibiscus sabdariffa* L. and its fractions against foodborne pathogen bacteria, as well as the viability of the application of the phenolic hibiscus extract as a natural meat preservative.

## 2. Materials and Methods

*Hibiscus sabdariffa* L. dry flowers and beefsteaks were acquired from a local grocery store in Chihuahua, Mexico. Folin-Ciocalteu’s phenol reagent, formic acid, quercetin, caffeic acid, chlorogenic acid, protocatechuic acid and Sephadex LH20^®^ were obtained from Sigma-Aldrich (Steinheim, Germany); gallic acid, water and HPLC-grade methanol were obtained from Merck (Darmstadt, Germany); trypticase soy broth and trypticase soy agar were obtained from BIOXON (Mexico city, Mexico).

### 2.1. Phenolic Extraction

For in vitro assays, one hundred grams of ground hibiscus calyces was mixed with 250 mL of acidified water (acetic acid 1%, *v*/*v*)–methanol-acetone (15:30:50, *v*/*v*/*v*) solution to obtain the extract. The extraction was carried out in a cold ultrasonic bath (Pittsburgh, PA USA Fisher Scientific) for 30 min. The extract was then evaporated until dryness under vacuum at 36 °C, and the concentrated extract was dissolved with 30 mL of acidified water (acetic acid 1%, *v*/*v*)–ethanol (50:50, *v*/*v*) solution (HE, hibiscus extract). Ethyl acetate (1:2.5 *v*/*v*) was added to the concentrated hydro-alcoholic extract, and then two phases were obtained by the liquid–liquid extraction. Organic (OP) and aqueous (AP) phases were separated in a separation funnel and afterward rotoevaporated (Flawil, Swizterland, Büchi) under vacuum until dryness at 36 °C. Each phase was stored at 4 °C for fractionation and further analyses.

For in situ assays, one hundred grams of ground hibiscus calyces was mixed with 250 mL of acidified water (acetic acid 1%, *v*/*v*)–ethanol (20:80, *v*/*v*) solution to obtain the extract. No further fractionation was carried out for the in situ tests.

### 2.2. Organic Phase Fractionation

Chromatography column loaded with Sephadex^®^ LH-20 gel was employed to fractionate the organic phase (OP). Nine milliliters of OP, obtained from liquid-liquid fractionation, was applied to the column previously conditioned with acidified water (acetic acid 1%, *v*/*v*). The OP was eluted with three different solvent mixtures, and each 200 mL of mixture corresponded to a fraction: (1) acidified water (acetic acid 1%, *v*/*v*)–methanol (80:20, *v*/*v*) (F1), (2) acidified water (acetic acid 1%, *v*/*v*)–methanol (50:50, *v*/*v*) (F2) and (3) acidified water (acetic acid 1%, *v*/*v*)–methanol (20:80, *v*/*v*) (F3). Each recovered fraction was rotoevaporated until dryness at reduced pressure at 36 °C and then dissolved with acidified water (acetic acid 1%, *v*/*v*)–ethanol (50:50, *v*/*v*) solution. Fractions were stored at −20 °C until in vitro microbiological analysis. 

### 2.3. Solid Phase Extraction (SPE)

A solid-phase extraction (SPE) was used to obtain a fraction rich in anthocyanins (FRA). A Sep Pak^®^ Vac 35cc (10 g) tC18 cartridge was previously preconditioned with 50 mL of methanol and sequentially rinsed with water (acetic acid 1%, *v*/*v*). Three milliliters of AP was eluted with a sequence of 30 mL of the following four solvent mixtures: (1) water (acetic acid 1%, *v*/*v*), (2) ethyl acetate, (3) water (acetic acid 1%, *v*/*v*)-methanol (80:20) (FRA) and (4) water (acetic acid 1%, *v*/*v*)-methanol (20:80). FRA was evaporated under vacuum at 36 °C, recovered and finally stored at −20 °C for further analysis. 

### 2.4. Total Polyphenols Quantification

Quantification of total phenolic content was performed to the extract, OP, AP and fractions (F1, F2, F3 and FRA). Total phenolic content was calculated by the Folin-Ciocalteu test [17] with gallic acid as a standard, and the results were reported as gallic acid equivalents (GAE). The absorbance was measured with a Lambda 25 Perkin Elmer spectrophotometer (Waltham, MA, USA).

### 2.5. Polyphenols Characterization

Hibiscus brute phenolic extract (HE) and all the fraction`s phenolic profiles were analyzed by High-Performance Liquid Chromatography coupled to a Diode Array Detector (HPLC-DAD). HPLC-DAD analysis was carried out on an Agilent 1100 (Santa Clara, CA USA), using a Zorbax Eclipse SB-C18 column with a column oven set at 30 °C. The volume of injection was fixed in 20 μL, while the mobile phase for the HPLC analysis was solvent A: Acidified water (formic acid 0.5%, *v*/*v*), and solvent B: methanol at a flow rate of 0.4 mL/min. The gradient (min/% B) employed was: 0/10, 7/15, 23/30, 30/40, 45/60, 50/80, 55/100, 60/100, 65/10 [18]. 

### 2.6. Antimicrobial Activity (In Vitro)

Minimum bactericidal concentration (MBC) and minimal inhibitory concentration (MIC) were determined for the brute phenolic extract, OP, AP, fractions and commercial phenolic standards (quercetin, caffeic acid, chlorogenic acid, gallic acid and protocatechuic acid) in agreement to the M07-A10 method of the Clinical and Laboratory Standards Institute [19]. Strains of *Escherichia coli* (ATCC 25922) and *Salmonella enterica* serovar Typhimurium (ATCC 14028) Gram-negative bacteria and *Staphylococcus aureus* (ATCC 25923), *Listeria monocytogenes* (ATCC 19114) and *Bacillus cereus* (ATCC 11778) Gram-positive bacteria were employed to obtain MBC and MIC. The latter technique was modified to be performed in microplates. A total volume of 200 μL was filled in each well, of which 10% corresponded to a respective strain in saline solution, and the remainder corresponded to trypticase soy broth and the volume required to achieve a concentration between 10 to 100 mg/L of extract, fractions or phenolic compounds. Additionally, bacteria and blank controls (with no polyphenols added) were prepared. In order to avoid cross-contamination, the bacteria controls were inoculated separately. The incubation was carried out in microplates in an Isotemp 550D (Pittsburgh, PA, USA Fisher Scientific) incubator at 37 °C for 24 h under aerobic conditions. MIC was set for the absence of turbidity in the well. MBC was determined by employing the first three wells without bacteria growth. Then, wells content was inoculated onto trypticase soy agar plates at 37 ± 2 °C under aerobic conditions. Finally, a growth lower than 10 CFU (colony-forming units) was set as the MBC. All tests were performed in triplicate.

### 2.7. Antimicrobial Activity (In Situ)

Slices of 10 g of beef steak (6 × 5 × 0.7 cm) were sprayed with 250, 500, 750, 1000 and 1250 mg/L (gallic acid equivalents) of the hibiscus extract. Beef slices were kept in petri dishes at 4 ± 1 °C for 10 days until microbiological analyses. Microbiological analyses of mesophiles and psychrophiles bacteria count were performed in agreement with the Official Mexican Standard NOM-092-SSA1-1994 [20] in order to find the best concentration to carry out the shelf-life test. Antimicrobial activity was performed in triplicate for each concentration. Antimicrobial activity (in situ) was performed to obtain the capability of the different concentrations of hibiscus ethanolic extract to inhibit the common bacteria on meat under commercial-like storage conditions.

### 2.8. Shelf-Life Microbiological Analysis

After the in situ antimicrobial activity, the concentration that seemed to perform the best was used to develop the shelf life study under refrigeration conditions, which was performed by monitoring the microbiological growth over the sprayed steak (with the phenolic hibiscus extract) and compared to the non-sprayed beefsteak (control). The meat was cut into thin slices of 10 g (6 × 5 × 0.7 cm) for each piece. Secondly, the pieces of steak were sprayed with 500 mg/L (gallic acid equivalents) of the hibiscus extract, while non-sprayed steak slices were employed as control. Sprayed and control slices were then placed in Petri dishes at 4 ± 1 °C for 15 days, employing triplicates for each day of analysis (0, 3, 6, 13, 15). During the shelf-life test, the numbers of mesophiles and psychrophiles bacteria were quantified following the Official Mexican Standard NOM-092-SSA1-1994 methodology [20]. 

### 2.9. Color Evaluation

Color evaluation was performed to the steak slices along with the shelf-life test. Color evaluation was performed on the surface of the sprayed steak and control steak by a Konica Minolta CR-400/410 (Tokyo, Japan) chromameter. The colorimeter was calibrated with a white tile (X = 94.9, y = 0.3185 x = 0.3124) before each analysis. Color determination was performed in triplicates, and the meat color was measured in quadruple, recording as L* (lightness), a* (redness), b* (yellowness), C* (saturation chroma) and h (hue angle) for each single meat slice. The results of mean and standard deviations obtained for each parameter were statistically analyzed.

### 2.10. Sensory Evaluation

A sensory trial analysis was carried out to find if a significant difference occurred between the control and treated meat. Triangular sensory test and overall acceptability test were performed as described by Anzaldua-Morales [21]. The sensory test was carried out in triplicate with one day off between tests, employing beef slices of about 50 g for each sample. Meat samples were cooked in a pan at 80 °C with a small quantity of vegetable oil until the beef was well done. The sensory panel was conformed by 30 untrained panelists.

### 2.11. Statistical Analysis

Repeated measurements analysis was applied to shelf life and color results employing Statistical Software SSPS version 15.

## 3. Results

### 3.1. Polyphenols Characterization

**Figure 1:**
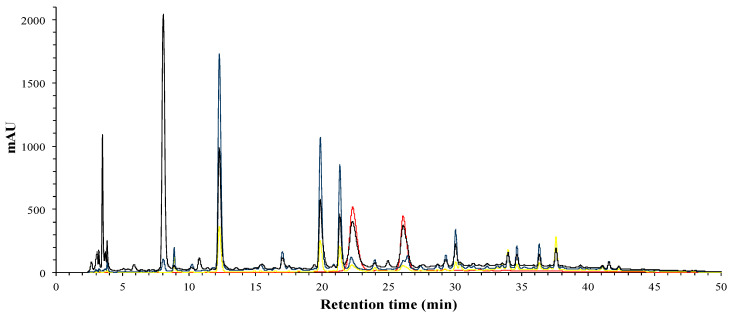
HPLC-DAD analysis of phenolic profile of hibiscus phenolic brute extract (HE); 280 nm (black), 320 nm (blue), 360 nm (yellow) and 520 nm (red).

The phenolic compounds of hibiscus extract, phases and fractions were identified by HPLC-DAD. Total phenolic compounds can be observed at 280 nm (Figure 1), while phenolic acids can be distinguished by its absorbance wavelengths at 280 nm and 320 nm, flavonols at 280 nm and 360 nm, and finally, anthocyanins at 520 nm.

**Figure 2:**
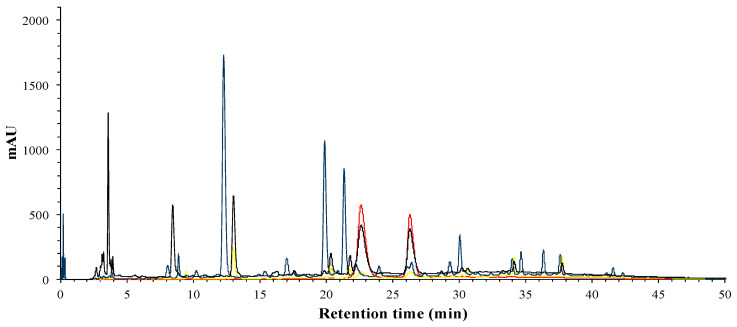
HPLC-DAD analysis of phenolic profile of the aqueous phase (**AP**); 280 nm (black), 320 nm (blue), 360 nm (yellow) and 520 nm (red).

After liquid–liquid extraction of the phenolic brute extract, the phenolic compounds were separated in organic and aqueous phase, according to the compounds’ polarity. In Figure 2, it can be observed that in the AP, the major compounds present, which are mainly phenolic acids and flavonoids, as well as the main anthocyanins previously reported in hibiscus flower, delphinidin-3-O-sambubioside and cyanidin-3-O-sambubioside [22,23].

**Figure 3:**
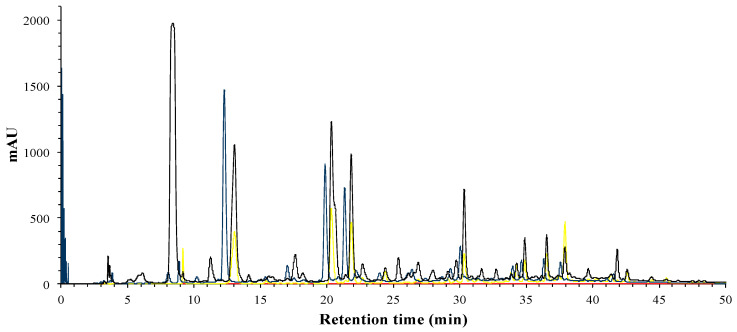
HPLC-DAD analysis of phenolic profile of the organic phase (OP); 280 nm (black), 320 nm (blue), 360 nm (yellow) and 520 nm (red).

Meanwhile, the compounds in OP correspond to phenolic and flavonoid families, as we can see in Figure 3. Anthocyanins were completely removed from the OP, as we can see with the absence of absorbance at 520 nm.

### 3.2. Fractionation

**Figure 4:**
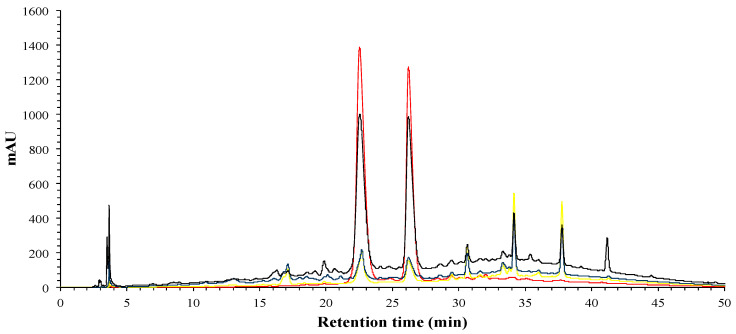
HPLC-DAD analysis of phenolic profile of fraction rich in anthocyanins (FRA); 280 nm (black), 320 nm (blue), 360 nm (yellow) and 520 nm (red).

A FRA (fraction rich in anthocyanins) was obtained from the aqueous phase by SPE in order to quantify the antibacterial effect of hibiscus anthocyanins. FRA phenolic profile (Figure 4) was analyzed by HPLC-DAD, the profile shows that the two anthocyanins (23 and 26 min) were the major compounds in the FRA, and secondly some flavonols.

**Figure 5:**
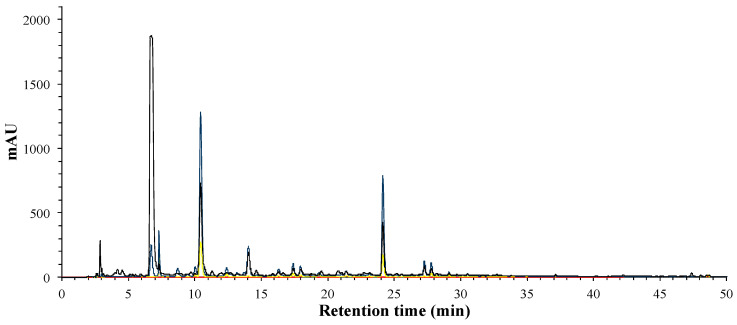
HPLC-DAD analysis of phenolic profile of the organic phase F1; 280 nm (black), 320 nm (blue), 360 nm (yellow) and 520 nm (red).

Organic phase (OP) was fractionated by low-pressure column chromatography; three fractions were obtained, and each was then analyzed by HPLC-DAD. The results acquired from F1 polyphenolic profile are shown in Figure 5, and it can be noticed that the major compounds present a maxima wavelength at 280 and 320 nm. The main peak with a retention time of 7 min presented a **λ** max at 280 nm and an m/z of 457 (in negative ionization mode) and remains unknown; the peaks at 10 and 24 min presented a characteristic UV-spectra of hydroxcynammic acids.

### 3.3. Antimicrobial Activity (In Vitro)

Antimicrobial activity was carried out employing different concentrations of hibiscus extract, AP, OP and its respective fractions. Thus, the initial concentrations of each solution were calculated by Folin-Ciocalteu methodology, employing gallic acid as standard. Concentrations of brute hibiscus extract and its different fractions are shown in Table 1. Total phenolic concentration of hibiscus calyces is 38.12 g/Kg d.b. in dry basis. Thus, this result is similar to what was previously reported by Sáyago-Ayerdi et al. [24] and Abou-Arab et al. [25], who report 36.5–45.5 g/Kg and 37.42 mg/g, respectively.

After the quantification of total polyphenols of each sample, aliquots were prepared in order to obtain different concentrations for MIC and MBC test. The results of MIC and MBC tests are shown in Table 2, where it can be seen that F1 presents the best MIC and MBC, followed by OP, the phase where F1 was obtained. However, F2 and F3 were obtained from the same fraction OP fraction and presented lower MIC and MBC than F1. Otherwise, the hibiscus extract, AP and FRA present similar values of MIC and MBC. This fact shows that anthocyanins are not responsible for the antibacterial activity of extract and AP, as was previously described by Borras-Linares et al. [26].

Gram-negative bacteria were more sensitive than Gram-positive bacteria to phenolic compounds in the present work. The former group present 25 mg/L GAE of MIC and MBC, while the latter presented up to 500 mg/L of GAE for MIC and more than 750 mg/L of GAE for MBC.

After obtaining the MIC and MBC of different extracts and fractions, standards of phenolic acids and flavonoids were tested in order to compare the results. The phenolic acids and flavonoids employed were chosen according to the compounds reported to exist in hibiscus flower and those that could present antibacterial activity (Table 3).

The results (Table 3) did not show that the phenolic commercial standards have presented activity against Gram-positive or Gram-negative bacteria, as was observed by Zhan and co-workers [27]. They observed that the Gram-positive Listeria monocytogenes bacterium was more sensitive to the rosemary extract, which was rich in phenolic acids content. Fraction 1 presented the highest antibacterial effect, and F1 is rich in phenolic acids; however, when tested separately, the four phenolic acids did not replicate the enhanced antibacterial effect of F1. Perhaps the synergy between all the compounds present in F1, the two phenolic hydroxycinnamic acids and the unknown compund with a m/z of 457 could be the cause of this enhanced antibacterial effect. 

### 3.4. Antimicrobial Activity (In Situ)

Antibacterial activity of the whole hibiscus phenolic extract (HE) was tested on the beef steak surface. Bacteria (mesophiles and psychrophiles) were quantified in meat slices sprayed with the phenolic hibiscus extract and in beef steak without hibiscus extract (control). Concentrations tested on the steak were between 250 and 1250 mg/L of HE and were stored at 4 ± 1 °C for 10 days. The results show that the control meat contained the major concentration of microorganisms with 3.46 × 10^9^ CFU (colony forming units)/g of meat of mesophiles and psychrophiles (Table 4). However, concentration of microorganisms was decreasing with the increase of hibiscus extract sprayed over the meat. Thus, at 750 mg/L of HE, the microorganism growth achieved the lowest count. On average, the values were 66 × 10^5^ CFU/g of meat of mesophiles and 1.26 × 10^5^ CFU/g of meat of psychrophiles. After this bottom level, at 1000 mg/L of HE, the microorganism growth presented an increase. At this concentration, colonies similar to yeast were observed; due to this growth, a Gram stain was performed to verify the presence of these microorganisms. In the last concentration tested, 1250 mg/L of HE, a decrease in the microorganisms count was observed again; however, different colonies were observed as in the 1000 mg/L of HE sample, which led to conduct a Gram stain for a second time, which showed that this growth was probably due to yeast and molds as well. Besides the growth of yeast and molds at high concentrations of hibiscus extract, the color in meat was considerably affected by adding the hibiscus extract. Therefore, at 750, 1000 and 1250 mg/L, meat color and appearance were affected, especially at 1250 mg/L, which appeared dry and cooked. This proliferation of yeast and molds could be due to the decrease of bacteria, which favors the yeast and molds to develop in higher concentrations of hibiscus extract. Thus, the concentration chosen to carry out the shelf life was 500 mg/L of HE, which decreased the microbial count and did not severely affect the meat color.

**Figure 6:**
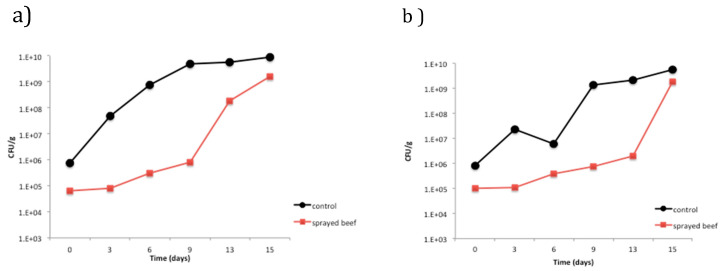
Shelf-life analysis for sprayed meat and control during a 15 days storage. (**a**) Psychrophiles and (**b**) mesophiles.

Shelf life analysis was achieved by monitoring the microbiological growth over 10 g of steak slices sprayed with 500 mg/L of the phenolic hibiscus extract and compared to the non-sprayed beefsteak (control). Microbial growth during shelf life test of 15 days showed a significant increase (*p* < 0.5) on mesophiles and psychrophiles, for both control and sprayed meat. Besides the significant microbial growth along the shelf-life time, a significant difference (*p* < 0.5) was observed between meat control and sprayed meat (Figure 6). The control meat thereby presented a higher concentration of mesophiles and psychrophiles microorganisms compared to the sprayed meat with HE. Besides, during the course of the microbiological study, the organoleptic properties of the control meat were changing. On day 9 of the analysis, the control meat displayed a bad smell and a microbial film over the meat surface. Meanwhile, the sprayed meat did not present a bad smell during the 15 days of the storage, but only at the end of this period of time did the meat show little spots of microbial colonies. 

Therefore, a potential increase in the meat shelf-life could occur as sprayed meat presented deterioration six days after the control meat. In other studies, an increase in the shelf life has been observed in meat covered with proteic films containing essential oils; however, those studies employed pathogen foodborne bacteria instead of the natural flora of the meat [28]. Besides, other studies on the beef shelf-life have shown that phenolic compounds are efficient as food preservatives [2]. Recent studies observed that a rinse with a hibiscus aqueous extract on hot dogs reduced the growth of foodborne pathogen as Listeria monocytogenes and methicillin-resistant *S. aureus* [12]. However, Higginbotham and coworkers [13] also observed that *S. aureus* and *E. Coli* presented a survival and proliferation in milk samples containing hibiscus aqueous extract, although this finding could be due to the protective effect of proteins on the bacteria strains.

### 3.5. Color Evaluation

**Figure 7:**
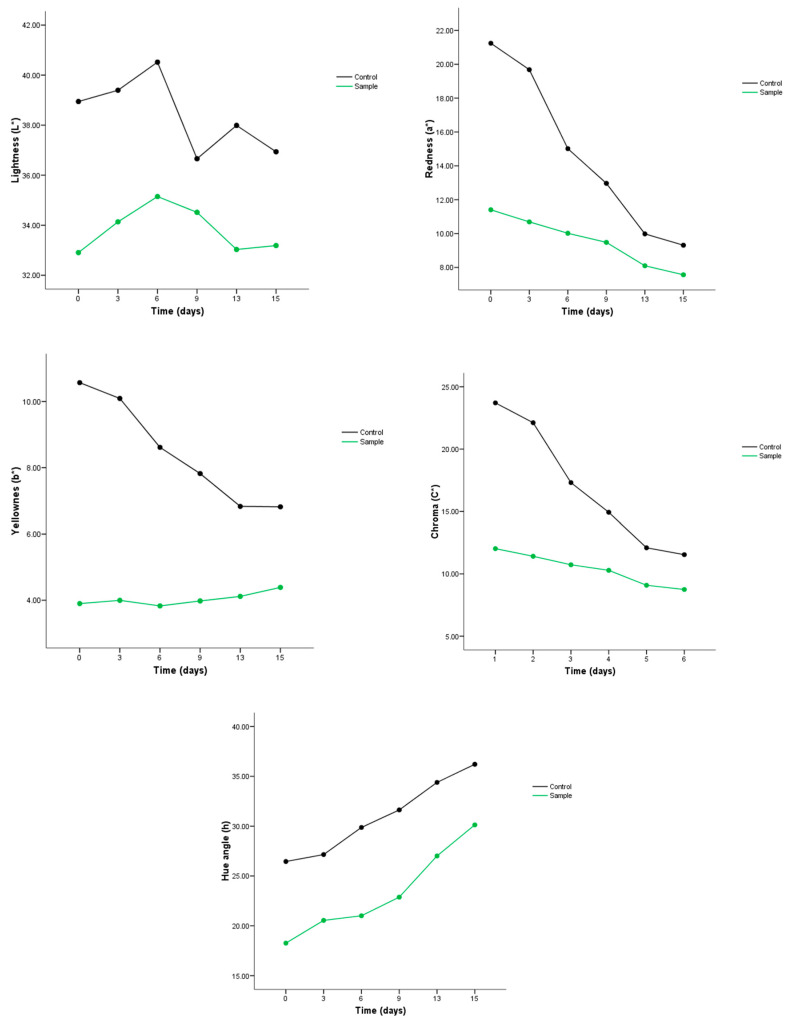
Color parameters for sprayed meat and control during 15 days of storage. L* (lightness), a* (redness), b*(yellowness), C* (saturation chroma) and h (hue angle).

A change in the sprayed meat was observed; thus, the steak slices presented an opaque color and a brown hue. Hence, along the shelf lifetime, both controls and sprayed meat showed a decrease in lightness (L*), the loss being bigger in the sprayed meat (Figure 7), while redness (a*), yellowness (b*) and chroma (C*) parameters had a similar trend as L* parameter. For all three of these parameters, the meat control presented higher values than the sprayed meat at the beginning and end of the analysis. However, the sprayed meat values presented more stability over time than control samples, whose values decreased significantly at the end of shelf life. For the redness parameter, an increase of values could be expected due to the anthocyanins color; however, an opposite result was obtained. Contrary to these results, the hue angle (h) displayed an increase during the shelf life measurements, while the control exhibited a higher angle than sprayed meat, although both types of samples values growing at the same rate. 

Similarly, Larrain et al. [29] found an increase of h value in lean tissue of bacon along 14 days of storage, the bacon came from pigs fed with porks feed with cranberry. However, they found no significant differences between L* and b* parameters in both the cranberry and control group during the shelf-life analysis. However, they found that a* and C* parameters decreased along the storage time, although the cranberry group displayed higher values than control samples. In another study, Zhang [27], found that L* and b* parameters increased in chicken meat with spice extracts, while for control samples, these parameters diminished. Besides, a decrease in the a* criteria was also observed, as was observed in the present work. 

### 3.6. Sensory Evaluation

For the sensory test, the sprayed meat with HE and non-sprayed meat (control) were cooked; after cooking, both samples presented the same appearance. However, the response of the triangular test over the two kinds of samples showed a significant difference, which means that hibiscus extract did change the taste of the sprayed meat. Panelists also found the meat a little more acidic than the control one; this could be due to the natural phenolic acids of the hibiscus extract and the acetic acid employed on the phenolic extraction process. 

Although the hibiscus extract did change the meat flavor, panelists did find the flavor pleasant, which is a favorable point on the sensory perception for consumers.

## 4. Conclusions

High doses of phenolic compounds are required to inhibit the growth and eliminate pathogenic foodborne bacteria, except for gallic acid, since it needed a concentration of 50 mg/L to kill *Salmonella enterica* serovar Typhimurium. In a different manner, a synergistic effect of phenolic compounds of *Hibiscus sabdariffa* extract might have occurred against bacteria tested in vitro for this work. On one hand, the organic phase F1, which was the richest in phenolic acids, presented the best performance against foodborne pathogen bacteria. On the other hand, the fact that hibiscus extract extended the meat’s shelf-life could be exploited as a natural food preservative, since the hibiscus flower is considered as a GRAS (generally recognized as safe) product. Besides, as the sprayed meat flavor presented a pleasing taste, and the color did not present a different appearance on the cooked meat, the application of hibiscus extract could be proposed as an antibacterial marinade for meat. This gives a healthy option to consume antioxidants and a natural alternative to increase the shelf-life of meat at cold temperatures. 

## Figures and Tables

**Table 1:** Concentration (mg/L GAE gallic acid equivalents) of extract and different phases and fractions obtained from Hibiscus sabdariffa L calyces.

	Concentration (mg/L) GAE
Brute hibiscus phenolic extract (**HE**)	15,250
Aqueous phase (**AP**)	12,700
Organic phase (**OP**)	13,500
Fraction 1 (**F1**)	3190
Fraction 2 (**F2**)	3290
Fraction 3 (**F3**)	3180
Fraction rich in anthocyanins (**FRA**)	4800

**Table 2:** Minimum inhibitory concentration (MIC) and minimum bactericide concentration (MBC) (mg/L of GAE) of hibiscus extract, phases and fractions over Gram-positive and Gram-negative bacteria.

	Gram-Negative				Gram-Positive					
	*E. coli*		*S. enterica* serovar Typhimurium		*S. aureus*		*L. monocytogenes*		*Bacillus cereus*	
	MIC	MBC	MIC	MBC	MIC	MBC	MIC	MBC	MIC	MBC
Hibiscus extract	300	500	300	500	200	400	200	400	200	300
**AP**	300	400	250	400	300	400	250	400	200	250
**FRA**	300	400	300	300	300	>500	300	>500	200	400
**OP**	125	200	150	200	125	150	125	150	90	100
**F1**	25	25	25	25	25	50	25	50	25	>75
**F2**	300	300	300	300	300	750	300	750	200	>500
**F3**	300	500	300	500	500	750	500	750	300	>750

**Table 3:** Minimum inhibitory concentration (MIC) and minimum bactericide concentration (MBC) (in mg/L) of phenolic acids and quercetin over Gram-positive and Gram-negative bacteria.

	Gram-Negative				Gram-Positive					
	*E. coli*		*S. enterica* serovar Typhimurium		*S. aureus*		*L. monocytogenes*		*Bacillus cereus*	
	MIC	MBC	MIC	MBC	MIC	MBC	MIC	MBC	MIC	MBC
Chlorogenic acid	>1000	>1000	>1000	>1000	>1000	>1000	>1000	>1000	>1000	>1000
Caffeic acid	>1000	>1000	300	500	>1000	>1000	>1000	>1000	800	>1000
Gallic acid	>1000	>1000	50	50	600	>800	400	>600	100	150
Protocatechuic acid	>1000	>1000	>1000	>1000	>1000	>1000	600	>800	500	700
Quercetin	>1000	>1000	>1000	>1000	>1000	>1000	>1000	>1000	>1000	>1000

**Table 4:** Psychrophiles and mesophiles count in different concentrations of HE on beef during ten days of storage.

	Psychrophiles CFU/g	Mesophiles CFU/g
Control	2.76 × 10^9^	1.86 × 10^9^
	3.46 × 10^9^	1.86 × 10^9^
	4.16 × 10^9^	2.20 × 10^9^
250 mg/L	1.09 × 10^8^	9.60 × 10^7^
	1.16 × 10^8^	8.70 × 10^7^
	1.09 × 10^8^	8.70 × 10^7^
500 mg/L	2.45 × 10^7^	5.80 × 10^7^
	2.15 × 10^7^	7.70 × 10^7^
	2.06 × 10^7^	7.10 × 10^7^
750 mg/L	1.38 × 10^5^	6.00 × 10^6^
	1.38 × 10^5^	6.00 × 10^6^
	1.02 × 10^5^	8.00 × 10^6^
1000 mg/L	4.04 × 10^6^	1.22 × 10^8^
	4.04 × 10^6^	1.22 × 10^8^
	1.09 × 10^6^	1.16 × 10^8^
1250 mg/L	6.35 × 10^5^	3.65 × 10^6^
	6.35 × 10^5^	3.65 × 10^6^
	8.07 × 10^5^	3.91 × 10^6^
